# Limb reconstruction in a resource-limited environment

**DOI:** 10.1051/sicotj/2021066

**Published:** 2021-12-31

**Authors:** Nando Ferreira, Sanjeev Sabharwal, Gamal Ahmed Hosny, Hemant Sharma, Ashok Johari, Vasudevan P. Nandalan, Mauro Vivas, Mangal Parihar, Selvadurai Nayagam, David Ferguson, Jan Duedal Rölfing

**Affiliations:** 1 Limb Reconstruction, Division of Orthopaedic Surgery, Faculty of Medicine and Health Sciences, Stellenbosch University 7600 Stellenbosch Cape Town South Africa; 2 University of California, San Francisco, Limb Lengthening & Reconstruction Center, UCSF Benioff Children’s Hospital Oakland 747 52nd Street, OPC 1st Floor Oakland CA 94609 USA; 3 Benha University Hospital 13511 Benha Egypt; 4 Hull Limb Reconstruction and Bone Infection Unit, Hull University Teaching Hospitals, University of Hull Hull HU3 2JZ United Kingdom; 5 Paediatric Orthopaedics, B. Nanavati Super Specialty Hospital 400056 Mumbai India; 6 Thangam Institute of Orthopaedic Surgery, Trauma & Ilizarov, Thangam Hospital Palakkad 678004 Kerala India; 7 Bone reconstruction and lengthening sector, El Cruce High Complexity Hospital 1888 Buenos Aires Argentina; 8 Center for Limb Lengthening & Reconstruction, Mangal Anand Hospital 400071 Mumbai India; 9 Royal Liverpool University Hospitals and Royal Liverpool Children’s Hospital L7 8XP Liverpool United Kingdom; 10 The James Cook University Hospital TS4 3BW Middlesbrough United Kingdom; 11 Children’s Orthopaedics and Reconstruction, Aarhus University Hospital Palle Juul-Jensens Boulevard 99, J801 8200 Aarhus Denmark

**Keywords:** Limb reconstruction, Limb lengthening, Limb salvage, Bone transport, External fixation, Ilizarov, Resource-limited environments, Low- and middle-income countries

## Abstract

*Introduction*: Limb salvage and reconstruction are often challenging and even more so in the limited resource setting. The purpose of this narrative review is to explore the strategies for addressing the unique obstacles and opportunities of limb reconstructive surgery in resource-limited environments globally. *Methods*: We review (1) the global burden and dimension of the problem, (2) the relevance of orthopedic forums and communication, (3) free and open-access software for deformity analysis and correction, (4) bidirectional learning opportunities, and the value of fellowships and mentoring between resource-rich and resource-limited countries, and (5) how societies like SICOT can help to tackle the problem. Finally, case examples are presented to demonstrate the choice of surgical implants, their availability in regions with limited resources, and how the universal principles of limb reconstruction can be applied, irrespective of resource availability. *Results*: Limb reconstruction can often be life-changing surgery with the goals of limb salvage, improved function, and ambulation. The contradiction of relatively few severe limb deformities in high-income countries (HICs) with abundant resources and the considerable burden of limb deformities in resource-limited countries is striking. Free, open access to education and software planning tools are of paramount importance to achieve this goal of limb reconstruction. Bidirectional learning, i.e., knowledge exchange between individual surgeons and societies with limited and abundant resources, can be reached via fellowships and mentoring. The presented cases highlight (1) fixator-assisted wound closure obliviating the need for plastic surgery, (2) open bone transport, and (3) hinged Ilizarov frames for correction of severe deformities. These cases underline that optimal clinical outcome can be achieved with low-cost and readily available implants when the principles of limb reconstruction are skillfully applied. *Discussion*: Limb lengthening and reconstruction are based on universally applicable principles. These have to be applied regardless of the planning tool or surgical implant availability to achieve the goals of limb salvage and improved quality of life.

## Introduction

**Figure 1:**
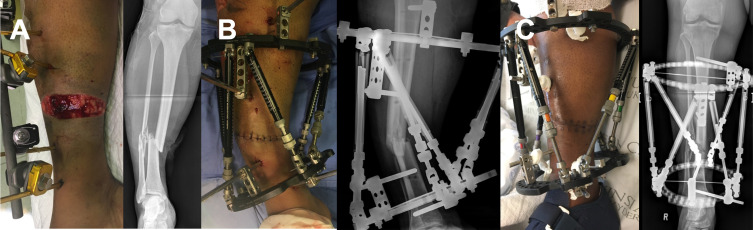
**Fixator-assisted wound closure of an open tibial fracture**. An open tibial fracture (A) was debrided and a hexapod external fixator is mounted in angulation to facilitate healing of the skin and soft tissue without plastic surgery (B). Bony alignment can be gradually restored thereafter (C).

Limb lengthening and reconstruction surgery, including the management of congenital and acquired deformities, complex acute fractures, pseudarthrosis, and osteomyelitis, have advanced substantially in the last two decades [1, 2]. Leaps in technological advances have introduced computer-aided deformity correction software and hexapod external fixators, intramedullary lengthening and bone transport nails, patient-specific additive manufacturing solutions, and a whole host of biological options to the field of limb reconstruction [3–7]. These technologies, however, are relatively expensive and not universally available, especially in resource-limited low- and middle-income countries (LMICs). In these settings, surgeons have to find innovative ways of using available technology and consumables to circumvent the lack of resources that may be readily available in high-income countries (HICs) [1]. One such example is deformity-assisted wound closure to manage open fractures with skin loss to compensate for the lack of plastic and micro-vascular surgical capabilities (Figure 1) [8, 9].

In this review, the key points of the lectures and case discussions of the SICOT PIONEER webinar: “Limb Reconstruction in a resource-limited environment” held on 15th October 2021 are presented. We have also supplemented cases and additional discussion, which could not be included in the webinar due to time constraints. The webinar is available in its entirety on SICOT’s YouTube channel [10].

## Global burden and dimension of the problem

There is a discrepancy between the distribution of wealth and resources and the prevalence of musculoskeletal deformities. While the majority of such deformities occur in LMICs, most research and treatment guidelines originate from HICs [11, 12]. Furthermore, treatment protocols and recent advances are often directed towards congenital deformities diagnosed at birth or recent injuries. Amputation is usually not accepted as a management strategy in many cultures, and access to high-quality prostheses is not readily available [13].

**Figure 2:**
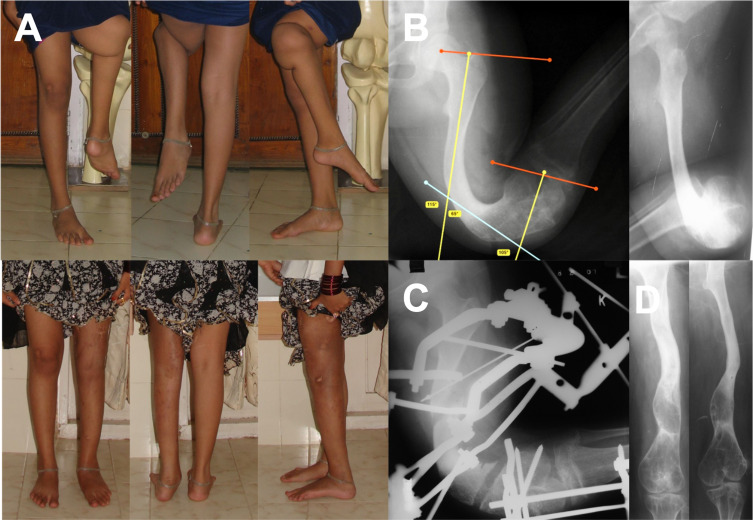
**Severe femoral deformity**. Clinical before and after images of a girl with a large complex femoral deformity (A). Preoperative planning (B) and meticulous execution with a hinged Ilizarov frame manipulating the double femoral osteotomy (C) resulted in an excellent clinical result (A and D).

In HICs, limb lengthening and reconstruction education focus primarily on correcting the mechanical axis deviation and preoperative planning using drawings and animations of simple deformities as examples. Unfortunately, most patients in LMICs present with complex multiplanar deformities (Figure 2). Consequently, surgeons need contextual education and training rather than simple preoperative planning techniques, including Sawbone- and cadaver-workshops. Appropriate clinical experience and long-term follow-up are vital in managing these patients with complex deformities. Perhaps, the limb lengthening and reconstruction community should revisit the conclusions derived from HIC based literature with the majority of papers reporting relatively small numbers of patients operated on by many surgeons over a long period and compare it to the vast experience with a large number of patients coming from countries with limited resources [1]. In HICs, computer-assisted external fixation and intramedullary lengthening nails are marketed as a breakthrough technology in limb reconstruction. However, studies report that in-depth experience with more traditional circular frames can avoid the expenses of computer-assisted external fixators and achieve very acceptable clinical outcomes for challenging cases [4, 7].

In conclusion, the incidence of untreated cases related to trauma and congenital deformities seems to be higher in resource-limited environments. Considering the experience developed in these areas may lead to a paradigm shift in managing posttraumatic and congenital deformities and opportunities for bidirectional learning.

## Relevance of orthopedic forums and communication

The limitations of managing patients with complex limb deformities with limited resources encompass knowledge, expertise, infrastructure, implants, implant quality, and affordability. This is contrasted by the large number of patients needing limb reconstruction and the surgeons’/trainees’ willingness to learn. The learning platforms frequently used are in-person meetings, email correspondence, videos (YouTube, Vumedi, OrthoTV, SICOT, etc.), social media such as Facebook groups, DocMatter, and instant messenger applications. While in-person meetings are expensive, opportunities for hands-on training (dry + wet labs) are essential for training purposes. In the developing world, email lists and group chat forums have become an informal form of sharing clinical experience and soliciting input from others for challenging cases.

Furthermore, relevant peer-reviewed literature can be shared, and such emails and group chats can be archived for future reference. Instant messaging is also widely accessible and easy to use but tends to focus on “image only” discussions and shorter types of answers. Video platforms have the advantage of building an archive over time. Video conferences come close to the interaction of face-to-face meetings but lack the option for hands-on training. Blended hybrid meetings that offer in-person and remote attendance options are becoming increasingly popular since the Covid pandemic.

In conclusion, there is an abundance of freely available information and online learning resources. Similar to scientific peer-reviewed papers, the quality of opinion is generally high, but expert opinions are predominant. Social media and instant messaging are also popular platforms. However, data security and patient-doctor confidentiality should be considered in such forums. Furthermore, no mechanism exists for quality assurance on advice given on these platforms. The popularity of web-based video discussions and hybrid congresses has also risen due to the COVID19 pandemic. A lesson learned from this period; hybrid congresses will likely be the norm in the coming years.

## Bidirectional learning opportunities and the value of fellowships and mentoring

The old paradigm of unidirectional knowledge transfer from “experts” in HICs teaching surgeons in resource-challenged environments to their peers in LMICs needs to be reexamined. Instead, a bidirectional learning model based on mutual respect, partnership, and synergy is more sustainable and can create opportunities to exchange unique knowledge and skills. For instance, high-volume surgeons with limited resources have unique clinical experiences and proficiencies relevant to their HIC counterparts-popularized as the concept of “reverse innovation” in health care [14]. Examples of such missed opportunities of bidirectional exchange include novel, cost-effective means of resource utilization and innovative solutions to address complex clinical problems, management of resurgent clinical entities (like polio-like conditions, chronic osteomyelitis, and tuberculosis), and the concept of “focus-factories” to improve clinical outcome and efficiency that are also relevant in HICs. Bidirectional international fellowships (fellowships provided to healthcare professionals from resource-constrained environments and fellowships hosted within resource-constrained environments) offer the ideal opportunity for combining expertise, pathology burden, innovation, and access to surgical solutions.

Professional societies such as SICOT, AAOS, POSNA, and OTA have established visiting scholars programs, where surgeons from resource-limited environments are supported to visit HIC centers for short-term clinical observerships [15]. These clinical opportunities are beneficial to the visiting surgeon and benefit their peers and patients back home [16]. Similar undertakings by industry and professional societies that support surgeons and researchers from HIC to visit and learn from facilities in resource-constrained environments will further strengthen international collaboration and help identify best management practices in both settings.

Barriers such as language, financial and cultural constraints and opportunities to improve the relevance of the clinical exposures for the visiting surgeon need to be breached [17]. Promoting free access to contextually appropriate educational material (such as Global Help: https://global-help.org/) and supporting remote learning opportunities using E-learning platforms can be very beneficial to enhance bidirectional learning further, strengthen academic partnerships and improve musculoskeletal care globally.

## Software for deformity analysis and simulation of corrections

**Figure 3:**
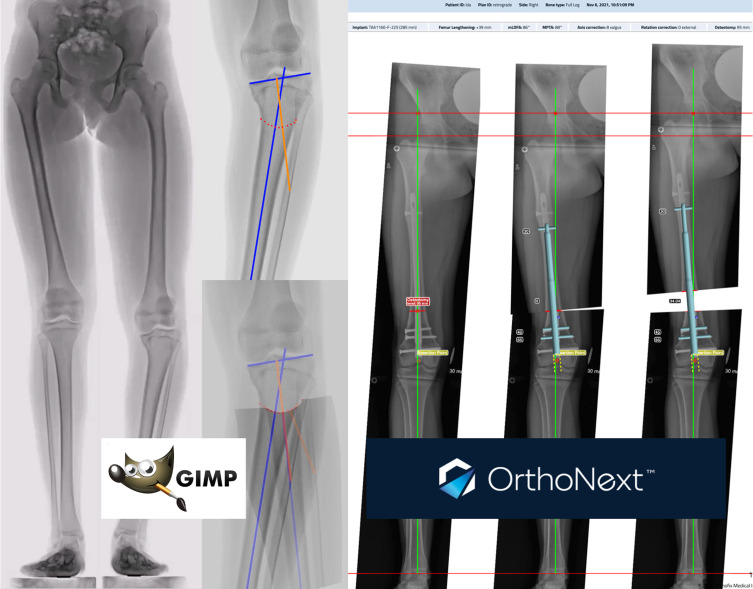
**Software for deformity analysis and correction**. *GIMP* is free, open-access imaging software. *OrthoNext* is also free and semi-automated.

Planning of limb reconstruction and deformity correction has evolved from paper-based methods to electronic software analyzing images with or without integration with picture archiving and communication systems (PACS). TraumaCAD (Brainlab AG, Munich, Germany), OrthoView (Materialise, Leuven, Belgium), OrthoNext (Orthofix, Verona, Italy), Bone Ninja (International Center for Limb Lengthening, Rubin Institute for Advanced Orthopedics, Sinai Hospital of Baltimore, USA) are some of the commercially available software options with comparable or higher reliability to the paper-based deformity correction [2, 18–23]. Furthermore, some of these applications offer semi-automated deformity analysis expediting the analysis and superimposing calibrated surgical implants (Figure 3).

GIMP (GNU Image Manipulation Program, https://www.gimp.org) is a free, open-access image editing software with powerful tools. It is available in 80 languages and on all major computer platforms. Calibrated radiographs can be analyzed after investing a short amount of time into learning to use the software and its tools [24, 25]. Deformity analysis, virtual simple and complex osteotomies like dome osteotomies, and multiple osteotomies can be performed. Superimposed images before and after the simulated osteotomy are also reassuring assets of this software (Figure 3).

Planning of deformity correction is crucial. The surgeon should obtain a first draft of correction, i.e., the perfect radiological correction, and then decide if locations of osteotomies are biologically feasible or if they can be adjusted to promote bone healing and early rehabilitation. If the first draft encompasses multiple osteotomies, consider if the number of osteotomies can be reduced. Structures at risk also must be considered and may warrant gradual rather than acute corrections. Intraoperative adherence to preoperative planning is essential. The anatomic and mechanical axes can be checked intraoperatively with reasonably simple and inexpensive tools [26, 27].

The choice of treatment/implant is affected by many factors, including cost, availability of implants, operating room accessibility, patient preference, host factors, and the need for in-patient stay and postoperative follow-up.

## Implant choice, cost, and availability

The armamentarium of limb reconstruction has expanded considerably, shifting treatment options from external to all internal treatment solutions. Here are some examples:

### Antibiotic coated interlocking nails

Advances in manufacturing have led to commercially available antibiotic-coated intramedullary nails. Off-the-shelf antibiotic-coated nails are a relatively modern solution for stabilizing fractures in an environment at high risk of infection like open fractures and non-unions [28, 29]. Gentamicin sulfate, for example, is contained within an intrinsically amorphous polylactide carrier, making it a bioresorbable coating. The surface coating prevents bacteria from adhering to the implant, with maximum effect over the first two weeks. For elderly trauma patients with tibial bone loss and where bone regeneration is less favorable, this implant can be considered to promote early rehabilitation. However, cheaper antibiotic coating of interlocking nails has also been described [30–32].

### 3D printed orthopedic implants

In recent years, 3D print of orthopedic implants, including titanium alloys, has become possible. Personalized 3D printed implants to reconstruct bone defects are on the rise. Despite their price and regulatory affairs, a growing body of literature shows the trend towards personalized medicine [33–35]. However, the role of 3D printed implants in limb reconstruction has yet to be defined, and innovative low-cost technology may become applicable on a large scale in resource-limited environments.

### All internal motorized limb lengthening and bone transport implants

Treatment of LLD and bone defects due to trauma, infection, or cancer is challenging. Motorized limb lengthening nails are costly but reliable devices to treat LLD [36]. All internal bone transport of long bones can be achieved with designated transport nails or Plate-or Fibula-Assisted Bone Segment Transport (PABST/FAST) [37–41]. The complication rate and patient comfort may favor these internal solutions compared with external fixation [7, 42]. However, the cost-effectiveness and clinical outcome has not been studied in detail using a robust scientific methodology. Furthermore, internal limb lengthening and bone transport devices harbor device-specific risks of complications and adverse events [43–50].

**Figure 4:**
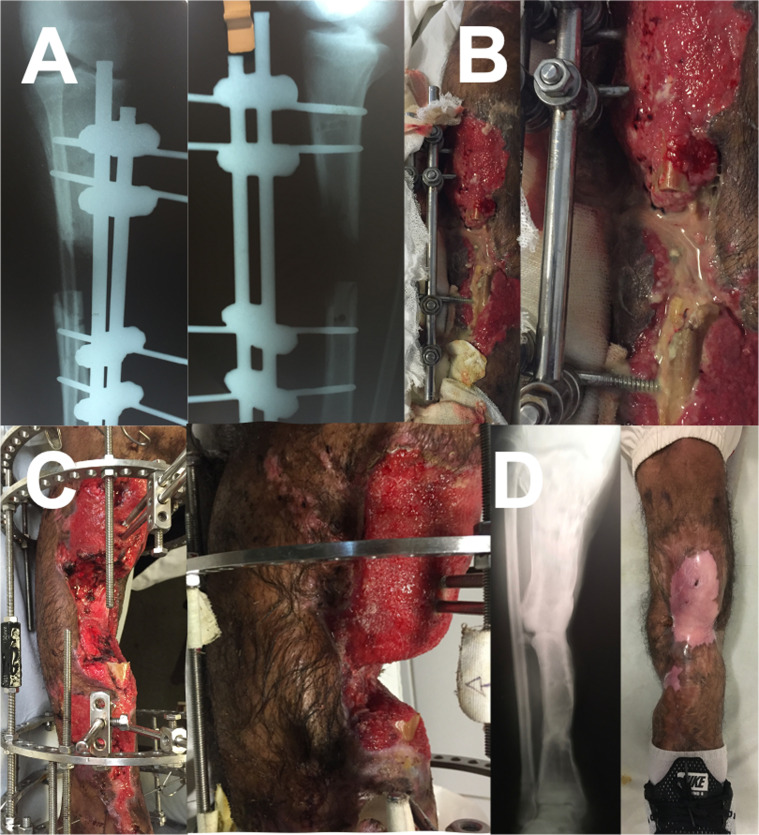
**Limb salvage**. A grossly infected limb with bone loss (A and B) was salvaged by distraction osteogenesis and transport of the soft tissue envelope with a simple Ilizarov frame (C). Bone healing and no infection were evident at final follow-up (D).

Finally, structures at risk, i.e., nerves and vessels, set biological boundaries for acute correction with internal devices. External fixators carry the risk of pin site infection, which can often be managed [51, 52]. With adequate planning and skillful execution, both open bone transport (Figure 4), as well as gradual correction of severe deformities (Figures 1 and 2), can be achieved at low-cost [53, 54]. These clinical cases highlight that even with limited resources, the principles of limb reconstruction can be applied and that these techniques truly can save limbs and change lives.

## The role of SICOT in tackling the problem

SICOT, as a global orthopedic body, is well poised to help tackle the problem of limited resources available for teaching and training contextually appropriate content to surgeons and trainees in LMICs. The problem, however, needs to be defined for a precise answer. Is it a lack of Education, Training, Innovation, Research or Resources or some combination of these factors? SICOT can be a knowledge broker facilitating knowledge and skill transfer by being a hub for organizing relevant training opportunities. Such activities can encourage innovation, promote knowledge creation by collaborative research, and help with the appropriate use of available resources through networking and advocacy.

SICOT has established a Limb Reconstruction Subspecialty Committee to acknowledge the subspecialty and foster education and training of SICOT members by defining the subspecialty curriculum, organizing lectures, courses, workshops and webinars, live surgery sessions, case discussions, fellowships and mentorships etc. Moreover, SICOT can encourage innovation by suitable incentives, e.g., awards and recognition. It can promote research with the possibility of multicenter collaborations amongst different members and centers, research education, mentorships, and publications. By facilitating networking and advocacy, SICOT can help garner valuable resources currently unavailable in LMICs. SICOT can also facilitate interaction with industry and enhance collaboration for education, training, innovation, research, and advocacy efforts.

There are unique ways of training and knowledge transfer, for example, by dealing with disasters, digital learning by virtual programs, blended learning combining digital with face-to-face learning, and the use of a Learning Management System (LMS), which brings with it the possibility of “on demand” 24 × 7 learning. Hence, SICOT can contribute substantially in disseminating knowledge, skills, and resources on the globe and propagating specialized knowledge for limb reconstruction.

## Concluding remarks

Limb lengthening and reconstruction is a demanding and labor-intensive surgical subspecialty. While the surgeons’ armamentarium has substantially increased during the last few decades, surgeons in resource-limited environments often have to be creative and cost-efficient when treating patients with highly complex deformities [8, 53, 54]. LMIC surgeons point out their complex and high-volume caseload but often have limited published data on their clinical outcomes. Little published work from LMIC, and reliance on direct knowledge transfer from HICs, means that outcomes and techniques to deal with complex problems are based on methods that have undergone less scientific scrutiny and analysis. High impact journals, mainly based and published in HIC, need to help address this inequity by encouraging scientific studies originating in LMIC. Bidirectional skills transfer would also benefit surgeons in HIC by developing skills to deal with complex and neglected problems. Brain drain from LMIC to HIC further complicates the already difficult situation.

Developing and freely sharing relevant techniques and technology should be embraced. Online learning platforms have accelerated during the recent Covid pandemic, which should be further harnessed. These learning tools will likely be part of future learning platforms. Even with online learning, LMICs are disproportionately affected due to lack of infrastructure, notably less reliable / slow internet connection. Preoperative planning is of paramount importance, and free software is becoming readily available. Free software, e.g., Gimp, OrthoNext, etc., is a welcome way forward, which will help and benefit a large proportion of LMIC surgeons and patients. Likewise, various video channels, email threads, and communication via social media has brought the information to the fingertip of surgeons, no matter where they practice.

The value of short-term observerships (2–4 weeks) without hands-on experience should be further assessed, as LMIC surgeons can spend large amounts of money with limited long-term benefits. Longer fellowships (6–12 months) with hands-on surgical experience can provide better knowledge and skills transfer but have limited availability. Hybrid fellowships suggested and being developed by the SICOT limb reconstruction subspecialty committee are one way forward, utilizing hands-on clinical experience in a single high-volume center and various other centers supporting a live telecast of surgery/supporting virtual case-based discussions/interactive sessions.

The role of societies like SICOT, POSNA, and various limb reconstruction societies worldwide should play a central role in developing relevant educational content. Professional associations can also facilitate and support bidirectional knowledge and skills transfer. They have the resources to identify the skills deficit, support the learning financially and help facilitate learning and flow of information.

Surgeons individually and in societies need to share and disseminate their skills and knowledge base. “Knowledge is a kind of wealth, the more you share, the richer you come” – Let us all strive to become rich.

## Conflicts of interest

***Nando Ferreira*:** Paid consultant for Orthofix Academy; Editor and Board member of the South African Orthopaedic Journal.

***Sanjeev Sabharwal*:** No conflicts of interest; Editorial board member – Journal of Bone and Joint Surgery (A), Journal of Limb Lengthening and Reconstruction.

***Mauro Vivas*:** Vice President ASAMI Argentina. No conflicts of interest.

***Jan Duedal Rölfing*:** Paid consultant for Orthofix Academy; Editor and board member of the Danish Orthopaedic Society.

***Ashok Johari***: President of SICOT. No conflicts of interest.

***Hemant Sharma***: President Elect British Limb Reconstruction society. Paid consultant for Orthofix, Smith & Nephew, Janssen (Johnson & Johnson) and Biocomposites. Editorial board Journal of limb lengthening and reconstruction, Member BOA research committee, Chair, SICOT limb reconstruction committee.

The remaining authors declare no conflicts of interest.

## Funding

This research did not receive any specific funding.

## Ethical approval

Ethical approval was not required.

## Informed consent

This article does not contain any studies involving human subjects. The depicted patients gave their consent.

## Author contributions

***N. Ferreira***, ***S. Sabharwal***, ***H. Sharma*:** Writing original draft, Reviewing and Editing.

***G. Hosny***, ***A. Johari***, ***D. Ferguson*:** Writing original draft.

***V. Nandalan***, ***M. Vivas***, ***M. Parihar***, ***S. Nayagam*:** Visualization, Reviewing.

***J.D. Rölfing***: Conceptualization, Writing original draft, Reviewing and Editing. Visualization.
